# Psychometric evaluation of a nursing competence assessment tool among nursing students: a development and validation study

**DOI:** 10.1186/s12909-022-03439-y

**Published:** 2022-05-16

**Authors:** Sheng-Miauh Huang, Su-Chen Fang, Chia-Tai Hung, Yi-Heng Chen

**Affiliations:** Department of Nursing, MacKay Medical College, No. 46, Section 3, Zhongzheng Rd., Sanzhi Dist., New Taipei City, 252 Taiwan

**Keywords:** Nursing students, Competence, Reliability, Validity, Instrument development

## Abstract

**Background:**

Nursing competence refers to the core abilities that are required for fulfilling one’s role as a nurse. A specific instrument comprehensively measuring competence among nursing students has not yet been developed. The purpose of the study was to develop and validate a nursing competence instrument for nursing students in bachelor training.

**Methods:**

A descriptive and explorative study design was used. Data were collected from students at one medical college in Taiwan in 2020 and 2021. A total of 241 nursing students participated in this study. We developed the initial instrument through systematic review, expert evaluations, and pilot versions. Its validity was then tested using confirmatory factor analysis (CFA) and criterion-related validity, while its reliability was tested using Cronbach’s alpha and test–retest analysis.

**Results:**

The final fit indexes of CFA were as follows: chi-square = 860.1 (*p* < 0.01), normed chi-square = 2.24, SRMR = 0.04, RMSEA = 0.07, CFI = 0.94, and TLI = 0.94. Cronbach’s alpha values for the subscales observed ranged from 0.91 to 0.98. The test–retest reliability coefficient for the Nurse Competence Scale was 0.515 (*n* = 30, *p* < 0.01).

**Conclusions:**

The instrument exhibited acceptable psychometric properties, thereby proving itself a valuable tool for evaluating nursing students’ competence at bachelor training. Further assessments of its reliability, validity, and generality from mentors’ and scholars’ views in different contexts and cultures are recommended.

**Supplementary Information:**

The online version contains supplementary material available at 10.1186/s12909-022-03439-y.

## Background

The concept of core competence was first defined as a harmonised combination of multiple resources and skills that distinguish a firm in the marketplace and therefore are the foundation of companies’ competitiveness in management theory [1]. Health care providers’ core competence is viewed as a combination of attributes, such as applied knowledge, skills, and attitudes that enable them to carry out care tasks efficiently and effectively [2]. The concept of core competency offers a common language for all health professions, defining expectations for optimal work [3]. Also, it is a promising way to reform and manage health-related education and ultimately improve quality of care [4, 5] and, in nursing, competency-based education provides a framework for nursing programs to train nursing students [6]. Fulfilment of competency-based education in nursing involves the recognition of core competencies, drawing plans of curricula and teaching programs that clearly express the attributes underpinning each core competency, and developing evaluation tools that offer a valid and reliable instrument to assess these core competencies [7].

Both generic and specific instruments have been developed to measure nursing competence during education among students and newly graduated nurses [8, 9]. The Nurse Competence Scale is the most widely used instrument measuring nursing competence [8], is rooted in Benner’s domains of clinical expertise [10]. Based on best-evidence synthesis, Hisar’s instrument for nursing students was recommended to assess nursing competence [11]. In Asia, Holistic Nursing Competence Scale was developed to measure general aptitude, staff education and management, ethical practice, the provision of nursing care, and professional development [12]. The competency inventory, a generic instrument for nursing students based on a learning perspective and developed by Taiwan’s researchers in 2013, measures five competency concepts: ethics and responsibility, general clinical skills, lifelong learning, clinical biomedical science, and caring and critical thinking reasoning [13]. Kao et al. [14] also developed Competence Scale for Clinical Nurses to measure three competency concepts: basic care skills, being dedicated to work, and patient-centered and ethical considerations. Specific instruments have been also developed for particular fields (e.g., mental health nursing; Moskoei et al. [15]) or for measuring certain competencies (e.g., cultural competence; Lin et al. [16]).

There is little consensus among educators on which core competencies to evaluate during nursing education programs or on the validated tools to use. There are significant problems associated with the language used to describe competencies when assessing nursing students [17]. Moreover, even though different nursing education programs have been established, little data is available about the evaluation processes and tools used for nursing students who experience classroom learning and clinical practice. The development of a holistic clinical assessment tool with a reasonable level of validity and reliability is needed [18]. Therefore, this study’s main aim is to develop and validate a nursing competence instrument for nursing students at a college/university in Taiwan.

Nursing students can acquire abilities through experience and learning and, subsequently, can develop competences (behavioural characteristics) [19]. Until now, there have been over 11,000 competence assessments [8]. In nursing, competence evaluation should be based on reliable and valid processes to capture the multi-dimensional nature of nursing competences [20]. Because there is no consensus regarding nursing competencies among nursing professionals, our literature review seeks to understand the initial concepts of nursing competency.

Both Matsutani et al. [21] and Fukada [19] identify the ability to understand individual situations as a main nursing competency. Hence, the clinical judgments toward health examination and many diseases and treatments was identified as medical-related knowledge and a basic ability to understand and assess the competence of individuals for nursing education in our study.

The nursing process including assessment, diagnosis, planning, implementation, and evaluation to deliver holistic, patient-focused care still guides nursing care today [22, 23]. In our study, we define basic nursing skill as the ability to apply nursing process in clinical practice. Here, caring, responsibility, and ethical concerns are involved in the nursing process [18, 24–26]. Additionally, critical thinking includes the processes of seeking, obtaining, evaluating, analysing, synthesising, and conceptualising information [27, 28] and is helpful in keeping nursing diagnostic processes accurate and reliable [29]. The ability to think critically among training nursing undergraduates has been described in several studies [18, 29]. We outline critical thinking as the ability to apply logical reasoning and speculation to respond appropriately to complex situations and problems nursing students face.

Poor communication and poor interpersonal relationships could be a central cause of bad patient outcomes and errors in clinical care [18, 30, 31]. Worldwide, leading healthcare organisations have recognised that interprofessional collaboration is indispensable to improve healthcare delivery [32]. So, it is expected that nursing students will appropriately express ideas and respond effectively to the patient when working in a medical care team. The growing diversity of cultures globally has intensified; thus, to deliver safe, effective, and culturally appropriate care, collaboration with global partners for the development of cultural competence in nursing students is one possible pedagogy [33]. Here, nursing students with global visions might have the necessary capabilities to face the challenges of emerging global conditions with an awareness of shared values and belonging to a common social and cultural space [34]. Life-long learning, recognised as a necessity for the development of the nursing profession [35, 36], is an important attitude of competency for newly graduated registered nurses [37]. With the advancement of health-related science and technology and the discovery of new diseases, we expect students to actively search for knowledge and understanding and to use these to meet their professional lifetime needs. This is consistent with the concepts regarding continuous learning or professional development in previous studies [11, 35, 36].

Although many core competencies have been studied in the past, the core competencies of nursing need to be revised in time to provide appropriate care as world health issues continue to change. Based on the above context of core abilities that are required for fulfilling one’s role as a nurse, nursing competence in our study was addressed specifically as medical related knowledge, basic nursing skills, communication and cooperation, life-long learning, global vision, and critical thinking.

## Methods

### Aim

The aim of this study was to develop and validate a nursing competence instrument for nursing students in bachelor training.

### Study design and settings

A descriptive and explorative study design was used to develop the new Nursing Student Competence Scale (NSCS), conceived to measure the ability of nursing students. Scale development and validation were conducted through a two-stage process at one medical college in Taiwan from August 2020 to July 2021.

### Participants

The participants were nursing students at MacKay medical college in Taiwan. The college enrolls 40 nursing students per year and has switched to 80 students per year beginning in 2018. Students who majored in nursing, who had finished at least one semester, and who could communicate in Chinese were invited for the study. Since previous temporary leave or drop out could influence the development process of nursing competence, we excluded students with these characteristics from the study. After the researcher explained the study purpose to all eligible students, those students would receive a paper copy of the questionnaire. Those students interested in participating in the study could complete and return the questionnaire in a stationary envelope.

### Instrument and procedure

#### Stage I: scale development

Item generation and reduction were conducted at this stage. Relevant concepts were first established via a literature review through which we identified core competencies among nurses or nursing students. Based on our literature findings, core competencies specifically refer to medical-related knowledge, basic nursing skills, communication and cooperation, life-long learning, global vision, and critical thinking. An exploratory qualitative study was then conducted using these concepts to collect data from 5 nursing professionals. Thus, nursing competence was addressed in the context of core abilities that are required for fulfilling one’s role as a nurse.

Initially, a pool of 30 potential items was generated through the aforementioned methods. The responses to each item were based on a 5-point Likert scale. A higher scale score meant a greater nursing ability. In our study, an expert’s rating and a pilot test were conducted to delete unnecessary items and refine the useful items. We recruited 5 experts from medical, surgical, obstetric, pediatric, or community nursing fields and asked them to rate the original 30 items of the new NSCS and discuss whether each item was relevant and important. Then, they listed the reasons why they revised certain items and gave specific suggestions. Each item was rated on two domains: relevance and importance. This rating was based on a 5-point scale. The higher the scale score, the more relevant or important it was. Finally, a 30-item NSCS was generated.

#### Stage II: scale validation

We used descriptive statistics (mean, standard deviation, frequency, and percentage) to describe the socio-demographic characteristics of the sample (age, gender, grade, private/ public high school, and family location) as well as to analyse item scores. The independent t-test was used to examine whether the difference between the highest (top 27) and lowest percentile (lowest 27) groups differed statistically (*p* < 0.05). Both the critical ratio (CR) of more than 3.5 and item total correlations of less than 0.30 were applied to reduce the number of items and discriminate the adequacy of each item from the subject response [38].

We assessed construct validity through confirmatory factor analysis (CFA). CFA analyses were performed using IBM SPSS Amos 21.0. CFA was performed using the robust maximum likelihood estimator method (MLR). Based on Hoyle’s [39] recommendations and a multifaceted approach to the assessment of model fit [40–43], chi-square (χ2), normed chi-square (CMIN/DF ≈2), comparative fit index (CFI; values ≥ 0.90), the Tuker and Lewis Index (TLI; values ≥ 0.90), the standardized root mean square residual (SRMR; values < 0.08), and the root mean square error of approximation (RMSEA; 0.05 ≤ values ≤ 0.08 indicate a good fit) are typically considered to indicate goodness of the model fit. The criterion-related validity was assessed by investigating its difference between junior (first and second year) and senior (third and fourth year) students. A 6-item measurement was used to assess each competence. Those six questions rated on a 0–100 scale (0 = “no competence” to 10 = “strongly competence”) were the criterion items in our study. A higher scale score meant a stronger competence (to be a competent nurse). We expected that a higher NSCS score would be associated with higher scores on the criterion questions.

The reliability of the nursing competence scale was evaluated using Cronbach’s alpha to assess the internal consistency of each factor and the overall scale. A coefficient greater than 0.70 was considered to indicate acceptable internal consistency, and coefficients greater than 0.80 were considered to indicate good internal consistency [44]. A test–retest analysis was carried out with 30 participants in their fourth year. Additionally, they were asked to complete the NSCS a second time within one month of the initial survey.

## Results

### Sample characteristics

During the study period, 246 students met the inclusion criteria. Of these, 5 refused to participate. The recovery rate was 98%. The 241 respondents ranged in age from 18 to 22 years (19.28 ± 1.01 years), and 82.99% were female. Table 1 shows their demographic details.

**Table 1:** Characteristics of the Nursing Students (*n* = 241)

Characteristic	n	%
Year		
First	67	27.8
Second	68	28.2
Third	76	31.5
Fourth	30	12..4
Sex		
Male	41	17
Female	200	83
Previous high school		
Private	41	17
Public	200	83
Family location		
North	176	73.0
Central	36	14.9
South	19	7.9
Eastern and outlying islands	10	4.1

### Validity

**Fig. 1:**
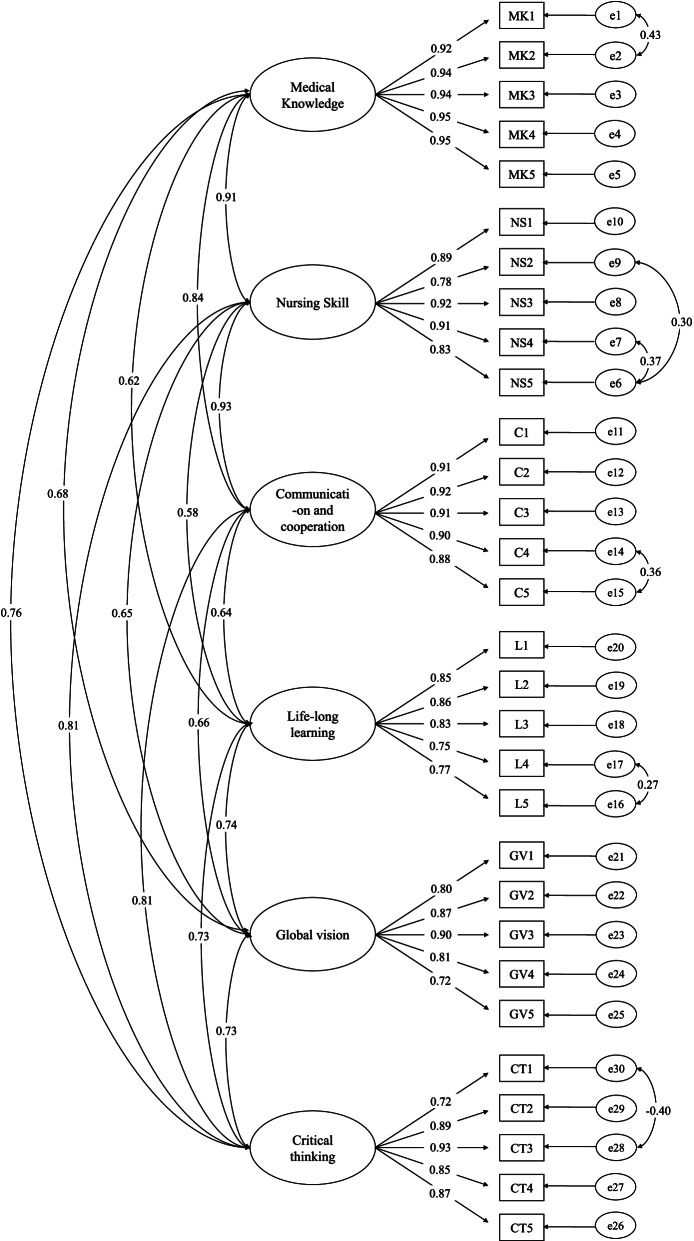
Confirmatory Factor Analysis of the Nursing Student Competence Scale

According to the item-level analyses, all items were kept and further analysed in the confirmatory factor analysis (Table 2). Table 2 shows the mean scores and the standard deviations of the six dimensions and the individual items. Further, we conducted CFA to verify 2 models. First, we performed a six-factor CFA without considering modification index (Table 3, model 1). Then, we checked the model 1 using modification indices when the value of the modification index was more than 10. Model fit indices are summarised in Table 3. Out of the 2 models, model 2 had the best model fit (model 1: SRMR = 0.04; RMSEA = 0.08; CFI = 0.93; TLI = 0.92; model 2: SRMR = 0.04; RMSEA = 0.07; CFI = 0.94; TLI = 0.94; Table 3). The model 2 results suggest that the six-dimensional model was the best model to be cross validated via CFA (Fig. 1).

**Table 2:** Item Analysis of Nursing Student Competence Scale (*n* = 241)

Competence/Items		Mean	Standard deviation	Critical ratio	Correlation to total Score
**Medical related knowledge**		15.81	5.25		
1	Can explain the meaning of the clinical examination	3.20	1.08	24.91*	0.846
2	Can explain the meaning of the laboratory test	3.18	1.11	27.62*	0.86
3	Can recognize the treatments and strategies of common diseases	3.25	1.06	24.52*	0.835
4	Can accurately assessment the effect of medical treatment	3.11	1.13	29.69*	0.854
5	Can manage the symptoms related to the alteration of patients’ condition	3.07	1.13	32.82*	0.865
**Basic nursing skills**		17.36	5.28		
6	Can stay of execution of basic nursing skills	3.18	1.23	33.34*	0.802
7	Can observe patients’ privacy and needs	3.87	1.06	27.07*	0.761
8	Can proceed physical assessment to confirm patients’ health issues	3.26	1.16	25.66*	0.851
9	Can provide individual care for patients	3.42	1.22	26.04*	0.848
10	Can have loyalty on nursing care	3.63	1.18	25.34*	0.793
**Communication and cooperation**		16.93	5.05		
11	Can provide clear and specific instruction on nursing care	3.35	1.11	26.64*	0.844
12	Can proceed effectively communicate to improve patients’ health problems	3.39	1.06	20.79*	0.814
13	Can fully communicate with family to improve patients’ health	3.49	1.07	21.31*	0.829
14	Can participate in care provided by multidisciplinary medical professionals	3.39	1.12	22.57*	0.846
15	Can help patients and their caregivers to identify the support and resources from different professional	3.31	1.10	21.30*	0.844
**Life-long learning**		19.44	3.32		
16	Can have attitude to active learning innovative knowledge	3.96	0.76	28.50*	0.661
17	Can take the initiative to attend courses promoting nursing professional	3.82	0.82	24.33*	0.642
18	Can face personal difficulties with self-reflection and find strategies for improvement	4.01	0.74	26.07*	0.643
19	Can effectively do time management to improve personal growth	3.85	0.76	25.18*	0.555
20	Can understand and plan self-development of personal nursing career	3.79	0.80	23.49*	0.629
**Global vision**		17.51	3.54		
21	Can understand the global trend of diseases, treatments, and health issues	3.51	0.78	19.31*	0.626
22	Can understand the innovative knowledge about nursing clinical practice and research	3.42	0.84	18.45*	0.74
23	Can understand the development of foreign and domestic nursing care	3.35	0.80	17.76*	0.711
24	Can perceive global and trans-cultural issues	3.48	0.80	18.96*	0.599
25	Can practice nursing work with multi-cultural perspective	3.76	0.91	23.14*	0.632
**Critical thinking**		18.00	4.18		
26	Can have ability of independently proceeding critical thinking	3.80	0.82	23.16*	0.62
27	Can use fundamental medical knowledge and logical thinking to revise care process	3.57	0.99	18.88*	0.782
28	Can assess health problems with consideration of personal different circumstance and provide appropriate nursing interventions	3.56	0.99	18.87*	0.836
29	Can arrange work with considering the priority of patients’ health problems	3.74	0.93	21.63*	0.785
30	Can manage patients’ health problem with application of the last evidence outcomes or creative thinking	3.33	1.02	19.96*	0.76

^*^p < 0.05

**Table 3:** Confirmatory Factor Analysis (CFA) fit indexes (*N* = 241)

	CFA index standard	Model 1	Model 2
Chi-square		999.9	860.1
DF		390	384
Normed chi-square (CMIN/DF)	≈2	2.564	2.24
RMSEA	< 0.08	0.081	0.072
SRMR	< 0.08	0.042	0.042
CFI	≥ 0.90	0.929	0.944
TLI	≥ 0.90	0.920	0.937

*DF* degree of freedom, *RMSEA* root mean square error of approximation, *SRMR* standardized root mean square residual, *CFI* comparative fit index, *TLI* Tuker and Lewis Index

Model 1: Maximum likelihood with robust standard errors; Model 2: Maximum likelihood with robust standard errors and modification indices

For the known-groups validity, our results showed that junior (first and second year) nursing students had lower NSCS scores than senior (third and fourth year) students (94.19 vs. 118.89; *p* < 0.01). The Pearson correlation coefficient between the total NSCS and the level of total criterion questions was 0.70 (*p* < 0.01). Modest and moderate correlations between each NSCS factor and each criterion question are shown in Table 4 (*r* = 0.270 to 0.705, *p* < 0.05).

**Table 4:** Correlation coefficients between criterion questions and Nursing Student Competence Scale (*N* = 241)

	**Criterion**						
	Medical related knowledge	Basic nursing skills	Communi-cation and cooperation	Life-long learning	Global vision	Critical thinking	Sum
**NSCS**							
Medical related knowledge	0.605**	0.705**	0.336**	0.424**	0.330**	0.514**	0.645**
Basic nursing skills	0.518**	0.654**	0.350**	0.431**	0.270**	0.485**	0.599**
Communication and cooperation	0.508**	0.638**	0.358**	0.427**	0.298**	0.457**	0.594**
Life-long learning	0.440**	0.430**	0.481**	0.523**	0.344**	0.470**	0.578**
Global vision	0.480**	0.500**	0.454**	0.532**	0.521**	0.505**	0.648**
Critical thinking	0.528**	0.541**	0.397**	0.448**	0.370**	0.533**	0.613**
Sum	0.596**	0.681**	0.443**	0.523**	0.396**	0.566**	0.702**

^**^p < 0.01; Abbreviations: NSCS, Nursing Student Competence Scale

The content validity index (CVI) of the NSCS across expert scores was 0.97 for relevance and 0.98 for importance. None of the final NSCS items was scored as irrelevant, unimportant, or inappropriate by the 5 experts. The CVI results were higher than the standard reported by Davis-a minimum CVI of 0.80 [45]. The findings indicate acceptance of the NSCS.

### Reliability

Reliability assessments included internal consistency and test–retest reliability. The Cronbach’s α coefficient for the 30-item NSCS was 0.98. Among the six factors, the Cronbach’s α coefficients ranged from 0.91 to 0.98. The factor-total correlations ranged from 0.75 to 0.92 (*p* < 0.01). The test–retest reliability coefficient for the NSCS was 0.515 (*n* = 30, *p* < 0.01).

## Discussion

This newly developed scale is a generic instrument designed to measure nursing students’ ability to meet various aspects of competence regarding nursing in Taiwan. The strength of this study is that the initial items were developed using a literature review and in-depth interviews with nursing professionals in Taiwan. The exploratory qualitative study pools 30 items for the six main concepts. Thus, we highlight the clinical practice backgrounds and competence needs among these nursing students in Taiwan. Compared to previous nursing competence instruments established by exploratory factor analysis in Taiwan [13, 14], we performed the validation study through confirmatory factor analysis, known-groups validity, and criterion validity based on our theoretical framework. Our results show that all six factors are representative and, hence, the newly designed Nurse Competence Scale has a good construct and criterion validity, indicating that it can be used to evaluate nursing students’ core competence. Internal consistency and test–retest reliability were used to assess reliability. The findings show the new NSCS demonstrated good consistency of results across items and measures from one time to another. Therefore, results from our study indicate that the NSCS possesses a substantial reliability and validity for assessing the core competence of nursing students.

Based on our results, the 30-item NSCS scale comprised six factors: (1) medical related knowledge, (2) basic nursing skills, (3) communication and cooperation (4) life-long learning, (5) global vision, and (6) critical thinking. Previous studies have reported that the main nursing competences may be divided into ability to understand people and situations, ability to provide people-centred care, and ability to improve nursing quality [19, 21]. Compared to these studies, the six nursing competences in our study correspond to the above three categories and, additionally, show the hierarchy among them (Additional file 1: Appendix 1). Our study shows that medical related knowledge is reflected in nurses’ ability to understand people and situations at the most basic level of nursing competences. Both basic nursing skills and communication and cooperation are classified as the ability to provide people-centred care, which is the middle level of nursing competence and provides foundational nursing care. Critical thinking, global vision, and life-long learning are categorised as the ability to improve nursing quality, which pertained to the highest level of nursing competence. As approximately half of the nursing competences in our study belonged to the highest level of nursing ability, our results reflect the nursing educators’ expectations of high levels of ability for the students in Taiwan. Issues around developing the nursing profession involve medical patriarchy in the health care system. When nurses demonstrate sufficient ability to improve the quality of care they provide, the nursing profession can be recognised by other health teams. Designing and arranging education courses tailored to different years in accordance with the development of nursing competencies are recommended for the future in Taiwan.

This study has some limitations which must be considered. All participants were enrolled from one university in Taipei. We did not survey nursing students at other facilities. This sampling bias might undermine the external validity of the results and cause selection bias. Whether or not the identified nursing competence in Taiwan are consistent with those of other colleges/universities merits further studies. Furthermore, nursing students who transferred from other colleges/universities were included in this study; we believe that some information from this group was meaningful. Because nursing competence is a linguistically and culturally sensitive measure, the applicability of the NSCS should be reappraised when used in different countries. Lastly, only thirty nursing students joined the test–retest measure. Further research is needed to assess this NSCS.

## Conclusions

This study contributes to a body of evidence about the psychometric properties of nursing competence. This validated study shows that the NSCS is an appropriate tool for measuring and assessing nursing competence among nursing students in Taiwan. Valid and reliable questionnaires can accurately measure the degree of development of each nursing competence. Misunderstanding the core competencies regarding nursing could cause nursing teachers to miss opportunities to assist students. Our results suggest that this NSCS scale should be integrated into bachelor nursing education in Taiwan to effectively assess the development of core competences among nursing students. The NSCS scale is useful for developing specific and effective strategies regarding the care dilemma in the teaching and learning environment and, here, a complete understanding of nursing competence will enlighten nursing professionals, especially nursing educators. More precise and specific teaching strategies are needed to overcome poor nursing competence in the future.

## Supplementary Information

**Additional file 1. Appendix 1.** Hierarchy diagram among competence, core competence, and curriculum design.

## Data Availability

The data that support the findings of this study are available from the corresponding author upon reasonable request.

## Abbreviations

CFA: Confirmatory factor analysis
CFI: Comparative fit index
CR: Critical ratio
DF: Degree of freedom
RMSEA: Root mean square error of approximation
MLR: Maximum likelihood estimator method
NSCS: Nursing Student Competence Scale
SRMR: Standardized root mean square residual
TLI: Tuker and Lewis Index
