# Supplementary figures and images for: Psychometric evaluation of a nursing competence assessment tool among nursing students: a development and validation study
Source: BMC Med Educ. 2022 May 16;22:372. doi: 10.1186/s12909-022-03439-y (PMC9109292; doi:10.1186/s12909-022-03439-y)

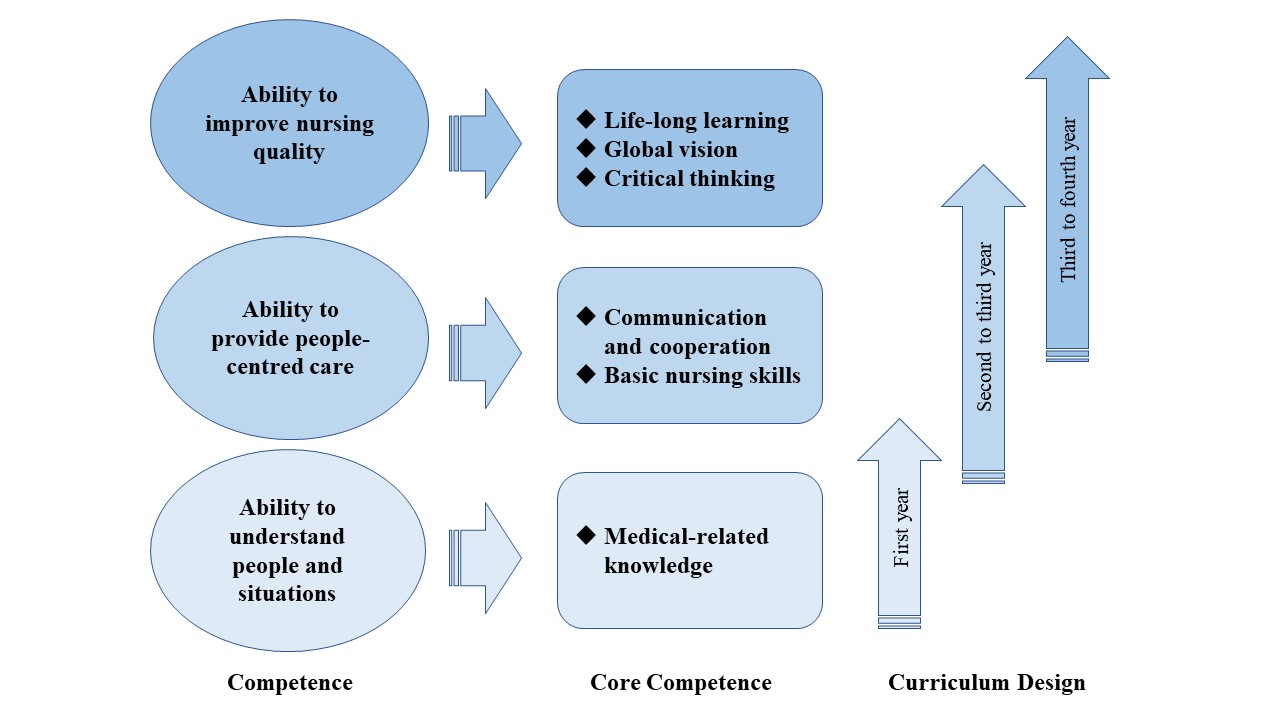

Supplement: Supplementary file 1 — Additional file 1. Appendix 1. Hierarchy diagram among competence, core competence, and curriculum design. [file 12909_2022_3439_MOESM1_ESM.jpg]
